# Gq Signaling in Autophagy Control: Between Chemical and Mechanical Cues

**DOI:** 10.3390/antiox11081599

**Published:** 2022-08-18

**Authors:** Inmaculada Navarro-Lérida, Anna M. Aragay, Alejandro Asensio, Catalina Ribas

**Affiliations:** 1 Molecular Biology Department and Center of Molecular Biology “Severo Ochoa”, CSIC-UAM, 28049 Madrid, Spain; 2 Health Research Institute “La Princesa”, 28006 Madrid, Spain; 3 Center for Biomedical Research in Cardiovascular Diseases Network (CIBERCV), ISCIII, 28029 Madrid, Spain; 4 Connexion Cancer-CSIC, 28006 Madrid, Spain; 5 Department of Biology, Molecular Biology Institute of Barcelona (IBMB), Spanish National Research Council (CSIC), 08028 Barcelona, Spain

**Keywords:** GPCR, Gq, autophagy, oxidative stress, mechanotransduction, extracellular matrix

## Abstract

All processes in human physiology relies on homeostatic mechanisms which require the activation of specific control circuits to adapt the changes imposed by external stimuli. One of the critical modulators of homeostatic balance is autophagy, a catabolic process that is responsible of the destruction of long-lived proteins and organelles through a lysosome degradative pathway. Identification of the mechanism underlying autophagic flux is considered of great importance as both protective and detrimental functions are linked with deregulated autophagy. At the mechanistic and regulatory levels, autophagy is activated in response to diverse stress conditions (food deprivation, hyperthermia and hypoxia), even a novel perspective highlight the potential role of physical forces in autophagy modulation. To understand the crosstalk between all these controlling mechanisms could give us new clues about the specific contribution of autophagy in a wide range of diseases including vascular disorders, inflammation and cancer. Of note, any homeostatic control critically depends in at least two additional and poorly studied interdependent components: a receptor and its downstream effectors. Addressing the selective receptors involved in autophagy regulation is an open question and represents a new area of research in this field. G-protein coupled receptors (GPCRs) represent one of the largest and druggable targets membrane receptor protein superfamily. By exerting their action through G proteins, GPCRs play fundamental roles in the control of cellular homeostasis. Novel studies have shown Gαq, a subunit of heterotrimeric G proteins, as a core modulator of mTORC1 and autophagy, suggesting a fundamental contribution of Gαq-coupled GPCRs mechanisms in the control of this homeostatic feedback loop. To address how GPCR-G proteins machinery integrates the response to different stresses including oxidative conditions and mechanical stimuli, could provide deeper insight into new signaling pathways and open potential and novel therapeutic strategies in the modulation of different pathological conditions.

## 1. Introduction

Cells are constantly exposed to a huge number of intra-and extra-cellular signals which requires a proper cell response for adaptation and homeostasis control [1]. Among the different sensors and integrators for such varying signals, the family of G-protein coupled receptors (GPCRs) arise as one of the most important transducers [2,3]. With over 800 GPCRs encoded in the human genome, this family of transmembrane receptors can bind a plethora of stimuli which include hormones, metabolites, and inflammatory mediators, influencing a diverse network of signaling pathways [4].

Apart from the classical chemical inputs, recent studies have begun to unravel the potential of mechanical and architectural properties of the environment as a new and alternative way to dynamically modulate cellular homeostasis [5]. Extracellular Matrix (ECM) is considered as an intricate meshwork of proteins and carbohydrates organized in a specific manner which acts not only as a reservoir of growth factor and bioactive molecules but also as a highly dynamic entity which provide mechanical rigidity and structural support to the cells [6].

All GPCRs contain seven transmembrane domains embedded within the cell membrane with several intracellular domains that trigger guanine nucleotide binding proteins downstream signaling pathways and, interestingly, extracellular domains in GPCRs have been reported as potentially critical elements in the interaction with components of the ECM and as a force sensing mechanism [7,8]. This opens the questions about the potential of GPCRs as linkers in the integration of all these signals (both chemo- and mechanical stimuli) raising the possibility that specific GPCRs and its downstream effectors can mediate the crosstalk between both types of triggers during the control of homeostatic feedback loops. Indeed, the ECM has the potential to significantly impact virtually on every physiological cellular mechanism [9,10]. Excessive deposition and increased stiffness of ECM has been directly linked with the progression of many different pathologies and has the potential to regulate cellular metabolism [11].

An important downstream process in the crossroad between chemo- and mechanosensing regulatory responses is autophagy [12]. The catabolic activity of autophagy is a fundamental cellular process that eliminates molecules and subcellular elements (including proteins, lipids, nucleic acids and even organelles) through a lysosomal-mediated degradative pathway for providing energy sources for ATP production or building blocks for protein synthesis [12]. Activated by different types of stress including those related with DNA damage, hypoxia, oxidative stress, inflammation, and food deprivation and by different challenges arising from mechanical (stretching, shear stress or hypotonic shock), autophagy can have both beneficial and deleterious effects [13,14,15]. Indeed, vascular and heart diseases, infectious diseases, neurodegenerative pathologies, and cancer have all been related to autophagy dysfunctions. Thus, autophagy represents a double-edge sword and for this reason the possibility to regulate autophagy in a time- and local-dependent manner represents a novel and valid therapeutic approach to control most of these disorders.

Several studies show the potential role of GPCRs as autophagy modulators through their downstream heterotrimeric G proteins [16,17,18,19] or through β-arrestin1 [20]. Interestingly, recent data from our laboratory demonstrate that Gαq can act as a general and relevant modulator of mTORC1 signaling over autophagy in response to fluctuations in different types of nutrients [21]; however, the contribution of mechanical forces and stromal remodeling impact in this regulation remains elusive. In this review, we will try to summarize the most recent advances in GPCRs, its connection with autophagy and the mechanisms underlying autophagic flux control. A better understanding of the interplay between autophagy and GPCR signaling networks will be very helpful to develop pharmacological strategies based on specific GPCR modulation with potential application to a great number of pathological situations, ranging from vascular and cancer to neurological diseases.

## 2. GPCRs Regulation and Functions, beyond the Classical Modulation

GPCRs share a common structural characteristic, the transmembrane region constituted by seven transmembrane spanning α-helices linked by three intracellular and three extracellular loops, together with an intracellular C-terminus and an extracellular N-terminus domain [7,22].

The GPCR superfamily has been subdivided into six classes based on how their ligand binds, or on their physiological function and structure: A, B, C, D, E and F. The classification considers amino acid sequences and functional correlation between species, with classes D and E missing in the mammalian system. Another analysis is based on the phylogenetic tree groups classifying GPCRs in five families: (G) Glutamate, (R) Rhodopsin, (A) Adhesion, (F) Frizzled/taste 2 and (S) secretin [23].

In all cases, upon activation by specific ligands, GPCRs undergo specific conformational changes allowing them to bind to heterotrimeric G proteins (α, β and γ subunits). This results in the activation of Galpha subunits by sequentially promoting the exchange of guanosine diphosphate (GDP) for guanosine triphosphate (GTP) to the heterotrimeric G proteins [7]. Then, GTP-bound G protein α-subunit dissociates from the βγ dimer, and then both of which bind to their respective downstream effector molecules. Recent findings further delineate complex receptor state transitions as transformations catalyzed both by G proteins and effectors that bind to the ligand-bound receptors [24,25]. This GPCR signaling can be terminated by the phosphorylation of the active receptor by specific kinases (GPCR kinases, or GRKs), followed by the binding of arrestin proteins which leads to GPCRs desensitization and internalization via clathrin-coated vesicle-mediated endocytosis [26,27,28]. Association of the GPCR with clathrin-coated pits induces its internalization and degradation through lysosomes [29] or alternatively, GPCR can be recycled back to the plasma membrane [30].

An additional aspect to be considered is that apart from the canonical signaling route from cell surface GPCRs and their downstream signaling partners, recent studies demonstrate that intracellular GPCRs can signal from internal cell compartments. They have been found at lysosomes, endosomes, endoplasmic reticulum, nuclei and mitochondria, displaying diverse cellular responses from their signaling at the cell surface [30]. This raises the concept of the existence of multiple signaling platforms that can be specifically activated by different stimuli. Thus, for some GPCRs, receptor activation and/or inhibition may occur at the cell surface; while for others, the fact that a ligand can get across the cellular membrane may change its functional response. From a pharmacological point of view, this opens the possibility of a new way to selectively target specific pools of GPCRs.

Common ligands for GPCRs are strikingly diverse: spanning ions, small molecules, lipids, peptides and proteins. Apart from its chemo-sensory function, recent studies have unveiled the participation of GPCRs in mechano-transduction [31,32,33,34]. Experimental evidence strongly supports the critical role of mechanical forces in the direct activation of these receptors. Mechanical stimuli can activate GPCRs without the involvement of their cognate agonists [35,36]. Supporting these observations, stimuli such as shear stress, hypotonic conditions and cell stretching, that alter membrane organization, have been reported as inducers of conformational transitions of GPCRs between an inactive to an activated state [35,37,38]. However, there are many aspects to be explored to further clarify how GPCRs might themselves be mechano-sensors and the mechanisms and functions behind this novel regulatory pathway.

Recent studies have shown that extracellular N-terminus within adhesive GPCRs can act as an anchor mechanism to the extracellular matrix (ECM), playing key roles in response to mechanical tension and in the control of their activity [37,38]. A recent revision approaches the different GPCRs which can be directly modulated by mechanical forces, highlighting the critical role of specific GPCRs in mechanotransduction such as adhesion GPCRs, APJ/apelin, AT_1_R, B_2_AR, B_2_R, ET_1_AR, GPR68, H_1_R, M_5_R PTH_1_R, all of them very relevant at vascular level [35,36,39,40,41]. Since most GPCRs contain at least one N-glycan chain in their extracellular domain, further investigation will be required to address their patho-physiological functions. As part of the GPCR network, heterotrimeric G proteins, such as Gαi and Gαq/11, seem to be the critical element in the orchestration of this mechanosensitive response [40,41]. A more detailed information is listed in Table 1 where GPCRs mainly coupled to Gq proteins are summarized. Adding to the complexity, mechanical forces can also be sensed by intracellular organelles [42]. Mechanosensitive organelles such as the nucleus, mitochondria or even lysosomes are also the residence for GPCRs and its downstream signaling, opening interesting new areas in GPCR field. 

## 3. New Avenues in Gαq/11 Signaling Complexes

Despite the high diversity of GPCRs, there are relatively small number of G proteins involved in the initiation of different intracellular signaling cascades. As we have mention, G protein α-subunits are defined by their ability to bind and hydrolyze GTP [77,78], which is a central event in their functionality. On the basis of sequence similarity Gα subunits have been divided into five different families (Gαs, Gαi, Gαq, Gα12 and Gv) [79]. Recent structures of GPCRs in complex with G proteins have revealed novel insights into G-protein coupling, including sequence determinants, and the flexibility of critical contact points (e.g., transmembrane helix 6, TM6) regulating G-protein access [80,81]. In this regard, the characterization of the structure of fully active GPCRs complexed with Gα proteins are being solved by advances cryo-electron microscopy techniques [82,83,84]. Several studies identify the N-terminus of Gα proteins as a key determinant of selectivity in GPCR binding and subsequent activation, providing new insights into the molecular basis of G protein-coupling selectivity beyond the Gα carboxy terminus [77].While some models propose a specific Gα binding to a particular GPCR, more recently it has been established the possibility that GPCRs can activate several Gα subtypes, displaying certain selectivity for specific isoforms [77,78,85]. 

The Gαq family of G proteins comprises four family members. The ubiquitously expressed Gαq and Gα11, Gα14 mainly expressed in liver, lung and kidney, and Gα15/16 (orthologues in mouse/human), specifically expressed in hematopoietic cells [86,87,88]. In this review we will focus on Gαq, the most widely studied member.

Classically, Gαq activity has been linked to the binding and activation of the β-isoform of phospholipase C, but in the last years a complex and important Gαq interactome has unraveled the possibility of activating different signaling pathways in distinct cellular scenarios [89]. Indeed, as intracellular GPCRs, it has become more evident that G proteins can dynamically be modulated to localize at diverse subcellular compartments. These new localizations provide novel mechanisms for signaling by G proteins [90,91]. A great number of cellular components have been reported to interact with Gαq, resulting in either propagation or deactivation of Gαq signaling. Gαq is known also to interact with components of the cytoskeleton, with important organizers of membrane microdomains, an also to reside in different organelles (see [79] for more details). This includes proteins involved in the regulation of GTPase activity such as GAPs (GTPase-activating proteins) and GEFs (Guanine-nucleotide-exchange factors) which led to the modulation of G protein cycle [92]. The regulator of G protein signaling (RGS) proteins act as GAP for G proteins, accelerating endogenous GTPase activity of Gα subunits. More than 20 members of RGS have been described with different members regulating Gαq activity [93,94]. Although RGS2 showed selectivity for Gαq/11 over Gi/o in vitro and in intact cell assay, recent data reveals new rules governing RGS-Gα recognition and the structural basis of this selectivity [95]. In a yeast two-hybrid screening using Gαq as bait, Ric8 has been also reported as a novel regulator and Gαq effector. siRNA-gene silencing of Ric8 shows a reduction in Gαq-coupled receptor-mediated ERK activation and intercellular calcium mobilization [96,97]. Ric-8 proteins were also shown to positively influence both plasma membrane localization and abundance of G proteins [98]. In this sense, an additional GEF-independent function for Ric-8 has been described during the protein synthesis process where it serves as a molecular chaperone that aids Gα subunit biosynthesis and mediates the initial association of G protein α subunits with endomembranes [99].

Interestingly, multitude of physiological processes regulated by GPCRs signaling regulators are involved in the rearrangements of the cytoskeleton with Rho GTPases as key. The signaling from the stimulation of GPCR to the RhoA activation is another important pathway which is mediated by Dbl-family GEFs [100,101]. Both G12/13 and Gαq/11 family members are upstream activators of RhoA. Recently, p63RhoGEF has been identified as a novel effector of Gαq involved in the stimulation of SRF-dependent gene expression. Biochemical and biophysical approaches have shown that p63RhoGEF directly and specifically associates with activated Gαq to enhance the guanine nucleotide exchange of RhoA, RhoB and RhoC [102]. In addition, Trio and Duet members, also act as Gαq effectors involved in the activation of RhoA [103]. Gαq interacts also with filamentous actin (F-actin) and moreover, the stimulation of Gq-coupled GPCR recruits tubulin to the membrane, both fostering PLCβ activation [104,105]. Thus, there is a cooperative relationship between cytoskeletal components, GPCRs and G proteins to confine signaling molecules in specific domains.

The general view of G protein signaling usually centers on its association with the cytoplasmic surface of the plasma membrane and the mechanisms underlying G protein cycling to carry out their cellular signaling functions. Plasma membrane (PM) is an extremely complex cellular entity, characterized by a two-dimensional asymmetric distribution formed by glycerophospholipids, sphingolipids, proteins, cholesterol, and carbohydrates. Its composition confers PM a specific fluidity, which enables the control of lateral diffusion and mobility of embedded molecules due to liquid-ordered and liquid-disorder plasma membrane microdomains [106]. Despite the controversies about the nomenclature, organization and dynamic of these microdomains considered as lipid rafts, what it is common to all these membrane nanodomains is its enrichment in cholesterol, sphingolipids, and specific anchored proteins [107]. Caveolae, represent a subset of membrane lipid rafts characterized by an enrichment in the membrane organizers caveolins and cavins. These two principal components have emerged as critical elements in the control of PM topography, rendering PM invaginations of 20–100 nm size which can undergo fusion endocytic and exocytic events through a variety of pathways ensuring protein recycling and chemical communication with the outside microenvironment [108,109]. Interestingly, plasma membrane nanoplatforms and its lateral organization have been proposed as critical driver modulators in the maintenance of membrane tension and in cellular mechanical responses [110].

Caveolins can act as scaffold proteins in multiprotein complexes, and they have been described as regulators of GPCR-Gαq system [111]. It has been reported the enrichment of GPCR signaling components in lipid rafts or caveolae, restricting their mobility and increasing their concentration, thus promoting the interaction and the initiation of different signaling pathways [112]. Although caveolae-lipid rafts seem to be a determinant of receptor-effector coupling, not all GPCRs (or G proteins) are found in this liquid ordered domains. Different types of G proteins appear to segregate differently with Gαq protein preferentially localizing into caveolae, while Gs and Gi isoforms are mainly localized into lipid rafts [113]. Consistent with this idea, it has been reported that Gαq but not Gs can immunoprecipitate with Caveolin-1 (Cav1). Interestingly, while Cav1 depletion does not alter Gαq subcellular localization, Gαq-mediated GPCR signaling is impaired.

The Cav1 contribution in the mechanosensing and adaptation in response to various mechanical stimuli, such as membrane stretching, hypoosmotic shock, shear stress or detachment [114,115], raises the possibility that Cav1-GPCR-Gαq could be a novel integrated module in the regulation of mechanotransduction. Indeed, it has been described that when cells are subjected to osmotic pressure, the enhancement of Ca^2+^ signals due to Gαq-Cav1 interactions is ablated [116] and more recently, an interesting work shows that the activation of Gq-calcium dependent signaling by mechanical stretch is mediated by the type of stretch and the amount of caveolae [117]. Previous studies have demonstrated that mechanical stressors can result in conformational changes in Cav1 to cause the release of key signaling molecules such as eNOS and Gαq [113,118].

In addition to Cav1, Gαq has been reported to interact with flotillins, lipid rafts resident proteins, in a mechanism which is nucleotide-binding independent. It has been demonstrated the implication of flotillins in Gαq-medated p38 MAPK activation, through Src family tyrosine kinase [119]. The interesting finding that Gαq can interact with both caveolin and flotillins opens the possibility of a differential regulation in each specific type of microdomains, both at plasma membrane and internal compartments. Furthermore, caveolin and flotillins can act as scaffolding proteins in signal transduction mechanisms directly connected with multiple cellular processes including the control of autophagy.

Interestingly while many other Gαq interactors have been reported, our group has recently described an unanticipated role of Gαq/11 as a key regulator of autophagy via modulation and interaction with components of the mTORC1 signaling hub. We have described that Gαq is part of the autophagic and lysosomal compartments participating as a general modulator of autophagy in response to serum, amino acids or glucose via interaction with the multifunctional p62/SQSTM1 protein [21]. In the next sections we will focus on autophagy emphasizing the potential and novel contribution of GPCR-G protein signaling in this process.

## 4. Autophagy between Nutrient, Mechanical and Oxidative Stress: An Emerging Role of Gαq

Autophagy is a highly conserved mechanism for cellular degradation in which cytosolic waste, protein aggregates and organelles are sequestered into a double-membrane vesicle (autophagosome) and delivered into lysosomes for breakdown [120]. Autophagy is orchestrated by sequential steps tightly control machinery in which ATG and associated proteins regulate the formation and maturation of autophagosomes into autolysosomes [121]. Under pathophysiological conditions, damaged or excessive accumulation of organelles such as endoplasmic reticulum, ribosomes, mitochondria, lipid droplets and peroxisomes can be degraded through mechanisms mediated by a collection of specific autophagy-related proteins [13,120].

Typically, autophagy stimulation depends on the mTOR system modulation [122]. It is initiated by the ULK1 complex (Unc-51-like-autophagy-activating kinase) which can receive input from cellular energy balance, and the availability of nutrients from mTORC1 (Rapamycin Complex1) and AMPK signaling networks (AMP-activated protein kinase). Canonical initiation of autophagy entails those metabolic stresses (chemical stimuli) such as nutrient deprivation. This causes mTORC1 dissociation from ULK1 which becomes active and binds to ATG13 and FIP200 triggering autophagosome formation [123,124]. In addition, mechanical stresses are also involved in autophagic flux control. Although it is unclear whether mechanical stresses may play a direct role in ULK1 activation, it has been reported that the mechanosensitive mTORC2 complex can indirectly induce ULK1 activation via inactivation of mTORC1 repressor function, through a FAK (focal adhesion kinase)-dependent mechanisms [125,126,127].

The extracellular matrix (ECM) constitutes a dynamic and plastic network of biophysical and biochemical factors that maintains tissue homeostasis. Changes in ECM composition, elasticity, and structure have also been reported to impact on autophagic flux raising the potential of matrix biology modulation as a critical controller of this process [9]. Apart from the interaction with physicochemical environmental imposed by the ECM, cells are also subject, and they have to respond to a great variety of mechanical forces due to other external forces, such shear stresses of fluid pressure (e.g., blood vessels), lateral stretches and compression (such in the case of muscles). Overall, this plethora of short- and large- scale forces elicit an adaptive cellular response in which autophagy seems to be a critical player.

In this adaptive response, cells can sense extracellular mechanical cues in different ways. This includes cellular adhesion complexes with ECM and/or cells, mechanosensors such as proteoglycans localized at the cell surface or mechanically activated ion channels (e.g., Piezo) [128,129] even plasma membrane-associated structures such cilium, caveolae, and clathrin-coated pits [115,130,131]. Moreover, multiple intracellular organelles, including autophagosomes, can also sense mechanical forces [132,133,134].

Mechanical cues imposed by forces or microenvironmental cues may affect the autophagic process through specific crosstalk with autophagy regulatory proteins (such as mTORC system, or AMPK pathway) or via mechanical regulation of cytoskeletal elements or phospholipid membranes, which can be crucial in the autophagic process [135,136,137]. Interestingly, a direct link between cell attachment to ECM and autophagy has also been reported [138]. Loss of cell attachment with the ECM usually results in programmed cell death via anoikis. In some cases, ECM-cell detachment can rapidly activate autophagy allowing for survival and re-attachment to the substrate [139]. However, it remains elusive the mechanism controlling this process with integrin-mediated adhesions emerging as a critical element [140]. Furthermore, ECM and integrin-mediated adhesion may trigger autophagy via FAK and ILK (integrin linked kinase) being relevant in different processes such as for immunosurveillance [141].

Additionally, matrix constituents have been shown to regulate autophagy in both directions, promoting or inhibiting this process, depending on matrix stiffness but also on its specific composition [142]. Indeed, recent studies have demonstrated an active and dynamic signaling role of specific extracellular matrix components on autophagic regulation which can act in both positive (activators) or negative (inhibitors) ways. Among them, decorin, collagen VI, kringle 5, endorepellin and endostatin function as activators and pro-autophagic matrix constituents that engage a diverse array of cell surface receptor for autophagic initiation [143,144,145]. In contrast, laminin α2 acts as an inhibitor. Thus, absence of laminin α2 permits excessive autophagy [146]. Interestingly, the action of all these components seems to be independent of the predominant nutrient concentrations. This unique class of matrix molecules can function as an alternative mechanism to the classical nutrient deprivation mechanism to safeguard cellular homeostatic balance through autophagic control and providing a new mechanism through which GPCRs could also be participating in the regulation of autophagy.

As we mention in a previous section, GPCRs are directly linked with mechano-transduction mechanisms (see Table 1). Supporting this idea, it has been demonstrated that mechanical perturbations can modulate GPCR conformational transitions [36]. Moreover, the response to shear stress can be directly modulated through the Gαq-coupled GPCR, GPR68 [35,41]. Interestingly, PKCζ, a protein that we have described as new interactor of Gαq [147], can be regulated by shear stress and activated by disturbed flow in atheroprone areas [148]. Further evidence on this mechanical stimulation comes from studies on adhesion GPCRs which display a long extracellular N-terminus with adhesive properties to the extracellular matrix or N-glycans modifications. These glycan chains have been reported to be able to be activated in the context of mechanical traction forces [38,149]. Further investigations are required to really address how these forces can structurally activate GPCRs in different contexts.

As a sequential process involving membrane remodeling events, autophagy is mechanically linked to cytoskeletal dynamics that lead to mechanical deformation and transport. Actin filaments and fibers and microtubule network can act as critical modulators in the control of organelle dynamics and autophagy control [150].

Although the most classical autophagy process relies on the delivery of cytoplasmic material to lysosomes via the double-membraned autophagosome, another form of autophagy, known as chaperone-mediated autophagy (CMA), occurs in the lysosome directly [151]. Thus, lysosomes can be considered critical hubs in the modulation of autophagic control. Lysosomes constitute a highly dynamic organelle which display important changes, including acidification and enhanced enzymatic activity. Furthermore, this organelle can move from the perinuclear localization to the cell periphery with important implications in cell metabolic control [152].

An emerging aspect to be considered is how lysosomal position can directly modulate its function. Lysosomes are transported bidirectionally through the microtubule network by dynein and kinesin motors, with microtubule motors such as dynein modulating the movement of lysosomes from the periphery towards a perinuclear location, while kinesins promote the scattering of lysosomes through the cytoplasm [153,154]. Recent evidence suggest that the distribution of lysosomes can be controlled by stimulation with different inputs. Under cell starvation, autophagosomes and lysosomes move toward the center of the cell facilitating the fusion of both compartments and the degradative capacity [155].

Several protein complexes have been implicated in the regulation of lysosomal positioning. One important regulator is the Rab7, a small GTPase involved in the coordination of appropriate coordination and homeostatic control of late endosomes and lysosomes [156,157]. Recently, WDR91 a Rab7 effector has been reported to be essential for lysosomal function [158]. In addition, the transcription factors TFEB and TFE3 are essential to promote the expression of multiple lysosomal genes. They play critical roles in the modulation of lysosomal biogenesis and distribution through the control of the lysosomal transmembrane protein TMEM55B (transmembrane protein 55B) expression [159]. TFEB acts as a link between autophagic process and lysosome biology [160], interacting with mTORC1 complex but not with mTORC2 controlling the lysosomal localization and function of mTORC1 [161].

Our recent studies strongly suggest that Gαq is a critical autophagy regulator raising the potential to control the shift between mTORC1-mTORC2 switch through lysosome control. We have recently reported a higher number of autolysosomes in cell lacking Gαq/11 compared to the wild type of counterpart which is directly linked with the involvement of this protein in the modulation of autophagy [21]. Immunofluorescence with LAMP1, a lysosomal-endolysosomal compartment marker, revealed that Gαq KO cells showed a predominant perinuclear lysosomal distribution in basal conditions, a phenotype that was mimicked by upon starving conditions. These results are consistent with a not-yet described role of Gαq in the modulation of lysosome dynamic regulation.

Furthermore, although many components of the autophagic machinery and autophagy receptors which are involved in the regulation of the process are being subjected to lysosomal degradation, in the case of Gαq, its presence in lysosomes does not alter its protein expression levels neither in basal nor in nutrient stress conditions which reinforce a critical role of this protein in the modulation of the autophagic control process.

**Figure 1:**
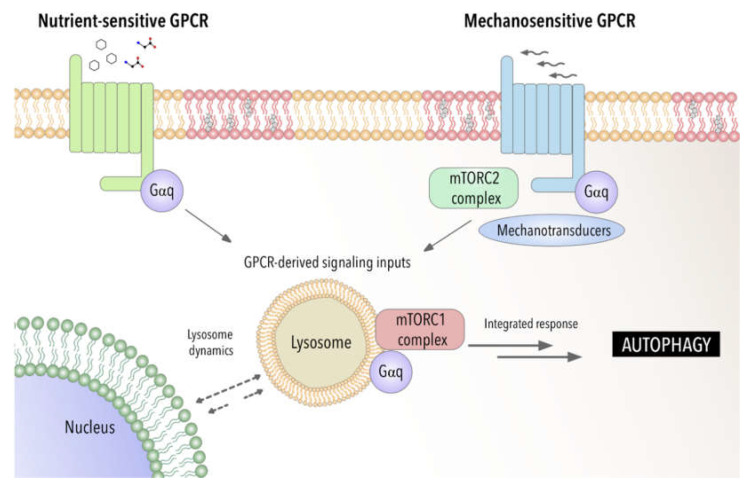
Gαq as a potential integrator of chemical and mechanical signals modulating autophagic process. Involvement of Gαq interactome-autophagy control in pathophysiological settings.

Recent studies have demonstrated by using a biosensor the presence of GTP-loaded Gαq/11 at endosomes [162]. This provides a powerful tool to be applied to other cellular organelles such lysosomal compartment to fully address the specific function of GPCR- Gαq signaling at these organelles. Furthermore, this raises the possibility that GPCR-Gαq signaling may act as a modulator of autophagy by acting as a switch between chemical and mechanical cellular responses (see Figure 1).

Importantly, in the last years there have been many reports that suggest that oxidative stress is also an important inducer of autophagy. Autophagy eliminates the toxic effects of reactive oxygen species (ROS) production to enable cell survival [163]. Reactive oxygen species (ROS) are produced in many cellular stress conditions such as hypoxia, nutrient deprivation or viral infection, among others. Under normal conditions, ROS levels participates in physiological processes regulating signaling pathways, to maintain cellular homeostasis. Excessive ROS production irreversibly oxidize organelles, proteins, lipids and DNA that can be partially counteracted by antioxidant enzymes, but high ROS eventually results in cellular damage by oxidative stress which is associated to the pathogenesis of diseases [164,165]. Autophagy serves the cell to clear off the damaged biomolecules and DNA produced by oxidative stress and there is a clear interplay between oxidative stress and induction of autophagy [166,167].

The main source of ROS in the cell (approximately 90%) is the respiratory chain in the inner membrane of the mitochondria. During oxidative phosphorylation the leaking of electrons from the electron transport chain (ETC) produce superoxide anion (O^2−^), hydroxylperoxide (H_2_O_2_), then OH^−^ under the catalysis of superoxide dismutase (SOD) and glutathione peroxidase (GSH-Px) [167]. Several works have established complex I and III as the major sites to generate ROS [168,169,170]. Under normal physiological conditions, ROS production is low, and the antioxidant machinery is able to scavenge ROS, while under high ROS production, oxidative stress occurs.

There are several studies that describe the crosstalk between oxidative stress and the autophagic machinery [165]. A direct link was shown through the inactivation of ATG4 by H_2_O_2_-oxidation to ensure autophagosome elongation [170]. On the other hand, ROS has been proven to induce autophagy through mTORC1 inactivation or AMPK activation [171,172,173] and through transcriptional regulation (HIF-1a, NRF2, p53 and FOXO3) [165,174]. These transcription factors mediate the induction of autophagy genes, including Beclin-1, LC3, p62, the mitophagy associated BNIP3 and NIX. On the other hand, ROS can regulate autophagy through the oxidation and inactivation of the ATG7, ATG10 and ATG3 proteins involved in the process of autosomal maturation and fusion to lysosomes and inactivate autophagy modulators like TFEB and PTEN [166,173,175]. Not only does ROS regulate autophagy, but autophagy can modulate ROS through the Keap-Nrf2 system. p62 can interact with Keap1 and release Nrf2 that translocates to the nuclei and activates antioxidant genes [176]. In this way, ROS can induce autophagy that in turn activates antioxidant genes to control ROS.

Extensive ROS can turn on the selective removal of damaged mitochondria by autophagy (mitophagy) [177]. Thus, autophagy limits the production of ROS and protects the cell from oxidative damage by selectively removing mitochondria [178]. There are several mechanisms of mitochondrial removal by mitophagy that have been described [178]. The most studied is the PINK/Parkin axis. Thus, mitochondrial damage triggers the translocation and regulation of the PTEN-induced putative kinase 1 (PINK1) and the ubiquitin E3-ligase Parkin. Parkin ubiquitinates several outer mitochondrial proteins that in turn recruit autophagy cargo receptors [179]. Among these receptors it has been described NDP52, optineurin and p62. The Rab proteins Rab5 and Rab7 located at the mitochondria surface and the RabGAP protein TBC1D15, that contains LC3 interacting domain, also help in this membrane recruitment process [180]. Several lines of work show that ROS stress leads to Parkin translocation to mitochondria to initiate the removal of mitochondria by mitophagy [15,181,182]. ROS accumulation can lead to disruption of mitochondrial membrane potential that stabilizes PINK. PINK1/Parkin mediated mitophagy has also been proven to play a protective role against oxidative stress in human nucleus pulposus cells [183]. ROS stress can also induce the translocation of adaptor proteins like DJ-1 to mitochondria [184]. The other mitophagy mechanisms described to induce mitophagy utilizes the mitochondrial adaptor proteins FUNDC1, BNIP3 and NIX [185]. These proteins localized at the outer membrane and can interact with LC3 to promote membrane contact and mitophagy [15]. The expression of several of these cargo receptors are induced by oxidative stress.

As mentioned before, ROS and mitophagy have a clear interplay and, although oxidative stress induces mitophagy, ROS can also lead to decrease mitophagy. The interaction between these processes requires a tight regulation and as we have seen both the mTORC1-p62 axis and mitophagy are crucial elements. Interestingly, recently it has been shown that mTORC1 signaling regulates mitophagy through the PINK1/PARK2 pathway [186]. In that context, the Gαq-dependent pathways may provide a mechanism of cross-regulation [79]. As stated Gαq is found in autophagic compartments and lysosomes and is part of the mTORC1 multimolecular complexes, contributing to inhibition of autophagy under GPCR activation and physiological conditions [21]. Gαq is also present at the outer and inner mitochondrial membrane [61] and contributes to cristae integrity and respiratory chain function. Interestingly, the absence of Gαq/11 alters mitochondrial crests and super respiratory complexes containing complex I and III which in turn may induce ROS production [187] As we have mention, Gαq also interacts with p62 and it is present in the autophagosomes [21]. Gαq is a key component for how nutrients and activated receptors sense and control autophagy, and through p62, can be an important component of the autophagy and oxidative stress control.

On the other hand, the role of Gαq in mitochondrial ROS generation is well sustained. As such, Gαq-induced cardiac decompensation has been associated with mitochondrial dysfunction and increased of ROS, with either enhanced expression of Gαq or activation of Gαq-linked GPCRs [188,189,190,191]. Noteworthy, the implication of Gαq-intracellular pathways via mitochondria and through mTORC1 and p62 seems to be a crucial asset to control the balance between oxidative stress and autophagy, but further work needs to be done to link these processes.

## 5. Autophagy in Disease for Good and for Bad: Gαq Involvement

Organisms have to constantly adapt to external stimuli and changes in their intracellular environment. Organs, tissues and cells have to face both chemical (e.g., Ca^2+^, amino acids, cytokines, chemokines and hormones) as well as physical challenges. Among the cytoplasmic responses to mechanical forces, recent studies have uncovered the role of autophagy in the translation of mechanical forces into biological responses [126,127,192,193]. Given the importance of autophagy regulation and dynamics of lysosomal system to ensure cellular fitness, it is not surprising that autophagy disruption can contribute to the development of several diseases such as metabolic disorders, cardiovascular or cancer diseases. Although the involvement of autophagy in these major diseases has been well studied (reviewed in [194], over the years autophagy regulation has grown in complexity and their consequences are less predictable. The importance of Gαq and Gαq-coupled GPCRs in all these contexts, together with its recently described importance in autophagy, strongly suggest that alteration in Gαq modulation signaling pathways can contribute to all these pathological situations. In this part of the review, we will focus on how autophagy may be involved in different pathologies, emphasizing as far as possible the influence of mechanical inputs.

Various metabolic disorders have shown functional defects in autophagy [195,196]. Since the lysosomal disposal of intracellular macromolecules leads to their breakdown into important metabolic intermediates, including amino acids, glucose, nucleotides, and free fatty acids (FAs), autophagy plays an important role in the response to energetic stresses, at both the tissue-specific and systemic levels [197]. Many studies have emphasized the importance of autophagy in conditions such as obesity, insulin resistance and diabetes that are characterized by metabolic alterations and intracellular stresses that have in common the accumulation of damaged cellular components. Silencing of ATG system promotes obesity and induces metabolic alterations [198]. Interestingly autophagy genes are differentially expressed and activated in a tissue and stage-specific manner. In general, nutrient limitation and different stress situations favor autophagy as a mechanism of cytoprotecting, reducing cellular death and limiting inflammatory response. Upon autophagy inhibition alteration of adipocyte differentiation, lipid metabolism and storing of lipids is drastically altered [199,200,201] For example, in obesity, autophagy is suppressed due to an increased in mTOR activity. Moreover, in patients with diabetes, changes in oxidative stress and autophagy have been reported [202]. Therefore, the enhancement of autophagy activity has been suggested as a novel therapeutic approach against organ failure associated to metabolic disorders.

In general, GPCRs regulate virtually all metabolic processes including glucose and energy homeostasis, particularly diabetes and obesity-related diseases. Several endogenous ligands such as free fatty acids and their receptors have been extensively studied in insulin secretion regulation, and glucose metabolism [203]. A growing number of GPCR are being identified as sensors of circulating of local concentrators of energy substrates or metabolic intermediates. Examples of these receptors include the amino-acid responsive receptors GPRC6A taste receptors type 1 members 1 and 3 (T1R1/T1R3), the calcium sensing receptor (CaSR) long chain fatty acid receptors GPR120 and GPR40, short fatty acid receptors GPR41 and GPR43 or hydroxy carboxylic acid receptors [204,205,206]. These GPCR nutrient receptors act via different G proteins including Gαq/11 and might be able to modulate the canonical metabolic regulators AMPK and mTORC1 [19]. In this sense, Gαq-coupled T1R1/T1R3 act as a direct sensor of the fed state and amino acids availability, leading to the activation of mTORC1 [207]. Nutrient and homeostasis fluctuations may also indicate the release of classical hormones and neurotransmitters that activated GPCRs, along a systemic regulation of autophagy. In this sense, β-adrenergic receptors activation has been related with autophagic flux favoring lipolysis [208], and hyperglycemia induces autophagy in pancreatic β cells through P_2_Y purinergic receptors [209]. In addition, drugs that target metabolic tissues have emerged as attractive diabetes therapeutic targets as well. The p62-mTORC1-autophagy axis has been described to regulate adipogenesis and energy control in a complex manner [210]. The potential and reported connections of Gαq signaling that we have described with this axis [21] may provide new insights in the mechanisms underlying these metabolic alterations. Recent studies have further confirmed the relevance of Gαq signaling for driving metabolic reprogramming in uveal melanoma [211] and in the regulation of glucose and lipid homeostasis [212] reinforcing a critical role of GPCR-Gαq system in metabolic diseases. Further investigation will be required to define the mechanisms involved.

**Figure 2:**
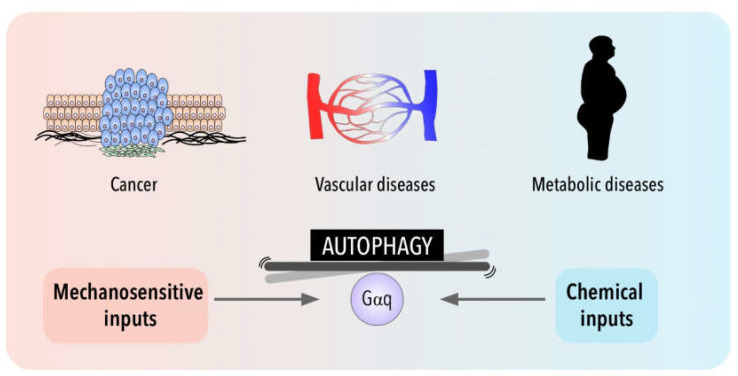
Gαq signaling and autophagy at the crossroads of a balance between mechanical and chemical cues and their impact in cancer, metabolic and cardiovascular pathologies.

Interestingly, cell metabolism is sensitive also to the physical cell microenvironment [213]. Although cell metabolism has recently emerged as one of the processes regulated by mechanical cues, the link between cell mechanics and metabolism is still poorly understood when compared with other pathologies such as cardiovascular diseases or cancer (Figure 2). Thus, in addition to metabolic intermediates, autophagy can influence metabolic reprogramming in epithelial cells through the involvement of mechanical forces such as shear stress [214,215] and, mechanical stretching/tension exerted by exercise has been shown to induce also autophagy in peripheral tissues (liver, pancreas and adipose tissue) [216].

The involvement of shear stress and mechanical forces in endothelial function has been well established and although, there is an increasing interest in the role of autophagic flux in vessel wall biology, the mechanosensors upstream of autophagy induction in endothelial cells are not well known [217]. Emerging evidence links alterations in autophagic flux with disease processes that include atherosclerosis, pulmonary hypertension and cardiovascular diseases [218]. Interestingly, a very recent study proposes a protective role of CMA (Chaperon Mediated Autophagy) against atherosclerosis [219]. Loss of autophagy may be a central mechanism through which risk factors elicit endothelial dysfunction. The role of autophagy in vascular development and in sprouting has been associated with a defective autophagy in mice lacking endothelial specific-TFEB factor [220]. The autophagic state of endothelial cells is also critical for vascular permeability [221]. Additionally, endothelial cells require autophagy to regulate tight junction proteins and maintain endothelial barrier integrity during inflammation [222]. Moreover, it has been reported that autophagy may be involved in the regulation of nitric oxide bioavailability, a crucial molecule that maintains vascular homeostasis in endothelial cells. Concomitant with a reduction in NO, loss of autophagy promotes and increase in endothelial ROS and inflammatory cytokine production [223,224]. Shear-stress-dependent autophagy is also important for NO production [224,225]. Indeed, in zones of low shear stress that are prone to develop atherosclerotic plaques, the impairment of autophagic flux induces endothelial NO synthase (eNOS) uncoupling, resulting in the production of superoxide instead of NO. Restoration of the autophagic flux favors the production of NO by endothelial NO synthase [226]. Interestingly, Gαq has been described as an important sensor of shear stress in endothelium [33,227]. Recent studies have demonstrated that changes in the type of flow can activate the same initial mechanosensing pathway involving Piezo1- and Gαq/11-mediated signaling with different atheroprotective response depending on the activation of α5 integrin, which is activated only by disturbed flow, but not by sustained laminar flow [228].

Regarding cardiovascular context, autophagy preserves cardiac structure and function under baseline conditions and is activated during stress, contributing to limit damage and preserve cardiac functionality during ischemia [229]. Cardiac cells are also subjected to tension. Several pathophysiological conditions, lead to an increase in cardiac workload and mechanical forces that are usually associated with pathological cardiac hypertrophy [230]. Mechanical forces can induce autophagy in cardiac cells being protective or detrimental depending on the context [217]. Indeed, during ischemia, autophagy has a protective effect on cardiomyocytes [231,232,233], while inhibition of autophagy improves cardiac function after reperfusion in an ischemia–reperfusion mouse model [232]. Much evidence places Gαq at the center of hypertrophic pathways in the heart (see [234], for more details). Indeed, Gαq signaling is both necessary and sufficient for the development of cardiac hypertrophy. The development of a cardiac hypertrophy phenotype has been correlated with a higher risk of heart failure [186]. Interestingly, it has become increasingly clear that both events are tied to the activation/presence of Gαq that can promote cardiomyocyte apoptosis and heart failure [235] by affecting vascular permeability and hypertension. Indeed, Gαq inhibition using specific drugs have been proposed to have anti-hypertensive role [236]. We have also described, in previous studies, a role of Gαq/PKCζ signaling axis in the development of cardiac hypertrophy in response to angiotensin II through a novel binding region on Gαq. Given the involvement of Gαq in cardiovascular function and in the process of autophagy, the potential participation of novel mechanisms downstream Gαq directly linked with autophagic flux in cardiovascular system is a relevant open question.

Autophagy is also recognized as a critical player in a context-dependent manner in cancer. Although it is well accepted that autophagy is important in many diseases, as described above, practically all clinical studies that involve autophagy manipulation are focused on cancer therapy. Autophagy networks are related to multiple aspects of cancer and may play a dual role with tumor-suppressive and tumor promoting functions depending on tumor cell type and stage [237]. Both inhibition of autophagy and its overstimulation are strategies tested in cancer, with the use of different drugs such as hydroxychloroquine, 3-methyl-adenina and everolimus as currently new strategies to be employed in clinics in combination with other chemotherapeutic treatments [238]. However, the high toxicity and adverse effects of these treatments urge a further understanding of the specific mechanisms by which autophagy modulates the different tumor progression steps.

Deficiency of autophagic genes has been found in various cancers. Impaired autophagy can promote tumorigenic environment through ROS dysregulation and inflammation processes [163,239,240]. On the other hand, at advanced cancer stages, increased autophagy can sustain tumor cell growth in nutrient-deficient, hypoxic tumor microenvironment and resistance to anoikis [241]. Upregulation confers chemoresistance and promotes the maintenance and survival of stem cell cancer status. Furthermore, autophagy inhibition can favor tumor cell invasiveness through the induction of de-differentiation mechanism. Thus, it seems that in premalignant lesions, enhanced autophagy might be beneficial preventing cancer, but in advance cancers most therapeutic strategies are focused on inhibiting autophagy [242]. Adding another layer of complexity, the novel Gαq role in modulating autophagy suggests that the balance between these processes characteristic of tumor growth might be altered in different cancer settings.

Furthermore, evidence identifies tumor microenvironment as a central driver of tumorigenesis in cancer [243]. Interestingly, cancer cells can also experience shear stress that can induce autophagy in different tumor cell lines [244,245,246,247]. Interstitial flow can promote the distribution of tumor-derived cells in primary tumor, while circulating tumor cells are also subjected to the shear stress from body fluids (blood, lymph and interstitial fluid) during metastasis [248,249]. It has been suggested that shear stress-induced autophagy can play an important role in controlling important cell responses from the regulation of cell size and metabolism to inflammation and cell death [217]. Moreover, the activation of tumor stromal fibroblasts to a state commonly known as cancer associated fibroblasts (CAFs) is critical. CAFs impact tumor progression by the modulation of multiple secretion functions of different factors (growth factors and inflammatory signals), by remodeling the extracellular matrix and even reprogramming their metabolism to provide nutrients and survival factors [250]. Moreover, autophagy can play a key role in CAFs activation [251]. Recent studies demonstrate that normal fibroblasts can differentiate into CAFs as protective responses to stresses under tumor microenvironment via the p62-Nrf2-pathway [252]. Furthermore, a molecular mechanism for CAFs activation has shown that tumor secreted lactate downregulates p62 in the stroma blocking AP-1-mediated p62 transcription [253]. Interestingly, we have described changes in the expression of Gαq affecting some partners such as p62 promoting its downregulation and favoring autophagic flux [21].

Altered GPCR pathways have increasingly been reported in cancer context and activating mutations in Gαq has been identified in approximately 80% of uveal melanomas. Considering Gαq as a component of the nutrient-sensing machinery, able to link nutrient availability with the activation of mTORC1 through its interaction with p62 [21] strongly reinforce its potential contribution in the modulation of both the tumor and its microenvironment, during tumor progression.

## 6. Conclusions

The highly conserved autophagy mechanisms are critical in cellular homeostasis by allowing degradation of cellular components in a lysosomal-dependent manner both in basal conditions or in response to internal or external fluctuations. In consequence, autophagy represents a central adaptation system.

Different studies have demonstrated that molecular mechanisms of autophagy are not only regulated by chemical stresses and metabolic challenges, such as starvation, but can also be modulated by mechanical stresses stemming from the environment. How these stimuli are integrated remains a challenge. In this review we propose a putative role of GPCR-Gαq signaling as a central integrator in this crosstalk suggesting its potential contribution as a balancer shaft (Figure 2). In addition to the canonical roles of Gαq and other heterotrimeric G proteins derived from their presence at the cytoplasmic surface of the plasma membrane, emerging evidence demonstrates that Gα subunit proteins can also localize in other cellular organelles such as endosomes, Golgi, ER, nucleus, mitochondria and most importantly in lysosomes. Determining whether such intracellular pools of heterotrimeric Gα protein subunits are dynamically generated via trafficking from the plasma membrane or represent resident stable subpopulations, as well as the identification of their location-specific interactors and functional roles, are active areas of research.

Furthermore, our recent study pointing out the critical involvement of Gαq as an autophagy regulator and the existence of GPCRs that can be directly modulated by physical forces providing new frontiers for deeper analysis. To understand how integration of chemical-mechanical-autophagic process occurs and the specific role of Gαq-GPCR in the interplay between metabolic cellular modulation and environmental cues will help us to understand the molecular basis of multiple diseases, in which autophagy represents a central element. Thus, a better comprehension of Gαq-mediated signaling pathways considering the context-specific modulation of autophagy will open new avenues for treating autophagy-related diseases based not only upon chemical but also mechanical inputs.

## Figures and Tables

**Table 1:** Gq-coupled GPCRs and their stimuli.

GPCRs (Coupled to Gq Protein)	Chemical Class of Natural Ligand	Mechanical Stimulation	References
5HT2A 5HT2B 5HT2C	Serotonin	Mechanical stretch	[43]
ADRA1A	Adrenaline/Noradrenaline	Shear stress	[44]
BB1	Bombesin	No reported	[45]
BLT1	Leukotrienes	No reported	[46]
CCK1	Cholecystokinin gastrin	No reported	[47]
CysLT1	Leukotrienes	Hypotonicity/ Increased intravascular pressure	[48,49]
EP1	ProstaglandinE2	No reported	[50,51]
ET1AR	Endothelin	Stretch	[34,52]
PAR1	Thrombin	Laminar flow	[53,54]
Gal2	Galanin	No reported	[55]
GHSR1a	Ghrelin	No reported	[56]
GnRH1	Gonadotropin	Insensitive	[57,58]
GRP39	Obestatin/Zinc	No reported	[59]
GPR68 GPR4	Protons	Shear stress	[41,60]
H1R	Histamine	Hypotonicity, direct membrane stretches, shear stress, intravascular flow	[34,58,61]
M5R M1R M3R	Acetylcholine Acetylcholine	Hypotoniticy and membrane stretch No reported	[62,63]
AT1R	Angiotensin	Hypotonicity, direct membrane stretch, pressure overload, increased intravascular pressure Pressure overload	[64,65,66]
MCHR	Melanin	No reported	[67]
B2R	Bradykinin	Shear stress, hipotonicity, Increase in plasma membrane fluidity	[68]
GPER	Estrogen	Mechanical stress	[69]
FFAR1	Fatty acids	No reported	[70]
PTH1R	PTH	Fluid shear stress	[71,72]
V1AR	Oxytocin Vasopressin	Stretch, shear stress	[34,73]
ADGRG2	No identified	Luminal fluid	[74]
P2YR	nucleotides	Fluid shear stress/Mechanical stress	[33,75,76]
